# Correlation between right atrial pressure measured via right heart catheterization and venous excess ultrasound, inferior vena cava diameter, and ultrasound-measured jugular venous pressure: a prospective observational study

**DOI:** 10.1186/s13089-024-00397-y

**Published:** 2024-11-29

**Authors:** Suppawee Klangthamneam, Krissada Meemook, Tananchai Petnak, Anchana Sonkaew, Taweevat Assavapokee

**Affiliations:** Faculty of Medicine Ramathibodi Hospital, Mahidol University, 270 Rama VI Rd, Thung Phaya Thai, Ratchathewi, Bangkok, 10400 Thailand

**Keywords:** IVC, RAP, RHC, uJVP, VExUS

## Abstract

**Background:**

Venous congestion is associated with adverse cardiovascular outcomes, necessitating accurate venous pressure assessment. Current methods, such as right heart catheterization (RHC), have limitations. Non-invasive techniques, including venous excess ultrasound (VExUS), inferior vena cava (IVC) assessment, and ultrasound-measured jugular venous pressure (uJVP), show promise but require validation in diverse populations.

**Aims:**

We aimed to assess the correlation between right atrial pressure (RAP) via RHC and non-invasive methods, including VExUS, IVC diameter with collapsibility index (CI) by American Society of Echocardiography (ASE) classification, and uJVP.

**Methods:**

In a prospective study involving 73 patients undergoing RHC, we evaluated the correlation between RAP and VExUS, IVC CI by ASE classification, and uJVP. We introduced and compared a modified VExUS grading system.

**Results:**

VExUS significantly correlated with RAP (p < 0.001), especially between VExUS grade 0 and grade 3. RAP significantly differed across IVC classifications by ASE (P < 0.001). VExUS grade 0 correlated with IVC class 1, and VExUS grade 3 correlated with IVC class 3. The modified VExUS grading system improved low and high RAP differentiation. uJVP exhibited a robust, highly significant positive correlation with invasively measured RAP (ρ = 0.67, P < 0.001).

**Conclusion:**

This study establishes a strong correlation between non-invasive ultrasound measurements (VExUS, IVC diameter with CI, and uJVP) and invasively measured RAP. These findings underscore the clinical potential of these non-invasive techniques in venous congestion assessment and patient risk stratification.

**Supplementary Information:**

The online version contains supplementary material available at 10.1186/s13089-024-00397-y.

## Introduction

Venous return and cardiac output are interdependent, as the venous and arterial systems function in series [1]. Venous return refers to the blood flow from the systemic venous network towards the right heart, and it holds significant importance in cardiovascular physiology [1–3]. The determinants of venous return, namely mean systemic filling pressure, RAP, and resistance to venous return, are crucial hemodynamic variables that greatly influence its dynamics [1–3]. Venous congestion in critically ill adult patients admitted to the intensive care unit is associated with an increased risk of mortality and acute kidney injury [4–8]. The conventional approach involves the invasive assessment of RAP through RHC. This procedure offers an approximation of mean systemic filling pressure and provides insights into venous congestion [1, 9, 10]. Regrettably, RHC availability is not widespread, and it carries inherent procedural risks, thereby limiting its repeatability.

Point-of-care ultrasonography assessment of IVC and uJVP have shown remarkable feasibility, reproducibility, and commendable accuracy in predicting elevated central venous pressures [11–14]. However, it is important to acknowledge that this approach has certain clinical limitations that should be considered [15, 16]. VExUS introduces a novel approach utilizing various measurements including the diameter of IVC, Doppler ultrasound assessment of venous flow through the hepatic vein (HV), portal vein (PV), and interlobar renal vein (IRV) [17]. By integrating these measurements, the “VExUS grade” can be determined, allowing for the identification of clinically significant venous congestion and the prediction of acute kidney injury in patients admitted to the intensive care unit [6, 7, 17].

While a study conducted in a Western population comparing the use of VExUS with RHC suggests a strong correlation between VExUS and RAP [18], to the best of our knowledge, there is currently a lack of similar investigations specifically in the Asian population. To bridge this research gap, we investigated the correlation between VExUS and invasively measured RAP via RHC, in relation to IVC diameter and its CI and uJVP, within the Asian population.

## Methods

Patients who underwent RHC for various indications at Ramathibodi Hospital between September 2022 and July 2023 were consecutively screened. Patients aged ≥ 18 years who provided informed consent were included in the study. Patients who were pregnant, required mechanical ventilation, or if their IVC, HV, PV, IRV, or internal jugular vein (IJV) were unable to be identified, were excluded from the study. The study received ethical approval from the Institutional Review Board of the Faculty of Medicine Ramathibodi Hospital, Mahidol University (MURA2022/429).

### Procedures and data collection

Inclusion criteria included participants aged over 18 years, scheduled for RHC, and willing and able to provide informed consent. Exclusion criteria comprised individuals who were pregnant, unable, or unwilling to provide informed consent, and mechanically ventilated patients. Patient demographic information, medical history, and echocardiographic findings were collected from RAMAEMR if they met the inclusion criteria. The selection of data elements for chart analysis was a result of collaborative decision-making among all coauthors. One member of the research team, a medical resident in their 3rd year who possessed a strong command of medical terminology, conducted the data extraction process. The evening prior to RHC, the research staff conducted a telephone confirmation with the scheduled patients and, on the morning of the procedure, within a 4 h window prior to the scheduled time, the research staff approached the patients to acquire informed consent. Upon obtaining consent, the research staff proceeded with the VExUS protocol, IVC maximum diameter and its CI, uJVP. The ultrasonographers involved in this study included an internal medicine resident and an internal medicine attending physician. The internal medicine resident underwent comprehensive training in VExUS, IVC, and uJVP through a combination of online modules [19], and hands-on sessions by the internal medicine attending physician. The internal medicine attending physician, who is also one of the researchers in this study, is an expert in ultrasonography and has received specialized training in VExUS, IVC, and uJVP. We utilized inter-rater variability to assess agreement among the examiners. Additionally, a pulmonologist and a cardiologist were part of the research team, providing expertise in image assessment and confirming the accuracy of grading. It is important to note that the ultrasonographers and researchers were independent of the clinical team.

### Blinding procedures

A member of the research team who performed the VExUS scans, IVC maximum diameter and its CI, and uJVP also participated in data entry, thus remaining unblinded to the results. However, the ultrasonographers documented the VExUS grading, IVC maximum diameter and its CI, and uJVP prior to the RHC procedure, and thus were blinded to the RAP data. Additionally, a clinician assessing RAP was blinded to VExUS grading, IVC maximum diameter and its CI and uJVP.

### VExUS scanning and IVC maximum diameter and its CI protocol

The VExUS scan was performed using the Philips Lumify phased-array probe and the Kosmos Torso Probe from Echo Nous. Patients were positioned in a reclined position with the head of the bed elevated at a 30-degree angle. The ultrasonographer initiated the scan by measuring the diameter of the IVC using the sniff technique, recording both the maximum and minimum diameters. The CI of the IVC was then calculated using the formula: CI (Dmax—Dmin) / Dmax. Measurements were taken approximately 2 cm from the junction of the IVC and the right atrium, or 0–1 cm caudal to the confluence of the HV and the IVC. The HV waveform was evaluated by placing the probe either in a subxiphoid position (with the transducer positioned 1–2 cm below the xiphoid process and the probe marker directed towards 12 o'clock) or in a coronal view (with the transducer positioned at the junction of an imaginary line extending from the xiphoid process to the midaxillary line and the probe marker directed towards the patient’s right axilla). Pulsed wave Doppler waveforms were obtained by placing the sample volume within the HV, at least 1–2 cm away from the junction of the HV and the IVC. The PV waveform and its pulsatility were assessed in the same area as the HV, using a coronal view. The Pulsed wave Doppler gate was positioned across the PV, and the Doppler scale was lowered to increase the waveform amplitude. This adjustment increased the accuracy in calculating the PV pulsatility index. The formula used was: PV Pulsatility Index = (Vmax—Vmin) / Vmax. To visualize the IRV waveform, the probe was maneuvered caudally and posteriorly while maintaining its position relative to the HV and the PV. Using color Doppler, the imaging box was centered over the renal cortex or renal pyramid to reveal the intrarenal vessels. The Pulsed wave Doppler gate was then positioned on the interlobar vessels to observe the waveform of the IRV [17].

**Fig. 1:**
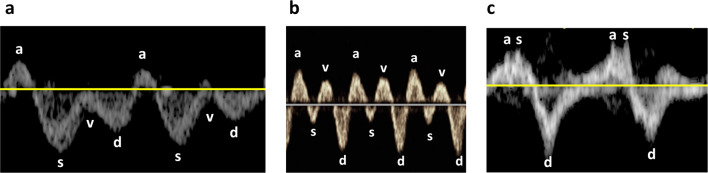
**a** Normal HV Doppler waveforms exhibit a small retrograde a-wave, followed by anterograde S and D waves, with the S wave being deeper than the D wave. **b** In mildly abnormal HV waveforms, the S wave is less deep than the D wave. **c** Severely abnormal HV waveforms show a reversal of the S wave, with only the **D** wave as a negative deflection wave; *HV* Hepatic Vein

**Fig. 2:**
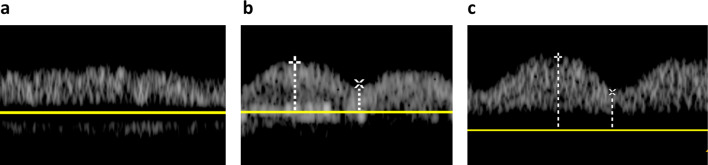
**a** A normal PV Doppler waveform demonstrates minimal pulsatility, with a pulsatility index < 30%. **b** A pulsatility index of 30–49% is considered mildly abnormal, **c** while a pulsatility index > 50% is classified as severely abnormal; PV = Portal Vein

**Fig. 3:**
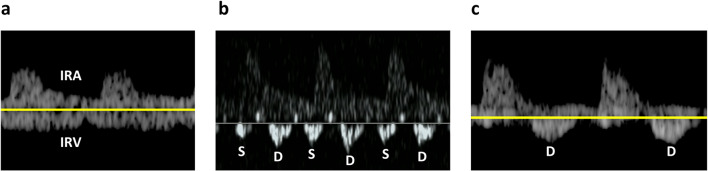
**a** A normal intrarenal Doppler pattern exhibits arterial pulsations generating a regular positive deflection wave, and IRV generating a continuous, smooth negative deflection wave. As renal venous congestion increases, venous pulsations become visible. **b** In mildly abnormal waveforms, venous pulsations with clearly visible anterograde S and D waves are present. **c** Severely abnormal waveforms display a retrograde S wave with only an anterograde D wave; *IRA* Interlobar Renal Artery, *IRV* Interlobar Renal Vein

The VExUS comprises evaluations of pulsed wave Doppler of HV, PV, and IRV. HV: Normal HV Doppler waveforms exhibit a small retrograde a-wave, followed by anterograde S and D waves, with the S wave being deeper than the D wave (Fig. 1a). In mildly abnormal HV waveforms, the S wave is less deep than the D wave (Fig. 1b). Severely abnormal HV waveforms show a reversal of the S wave, with only the D wave as a negative deflection wave (Fig. 1c). PV: A normal PV Doppler waveform demonstrates minimal pulsatility, with a pulsatility index < 30% (Fig. 2a). A pulsatility index of 30–49% is considered mildly abnormal (Fig. 2b), while a pulsatility index > 50% is classified as severely abnormal (Fig. 2c). IRV: A normal intrarenal Doppler pattern exhibits arterial pulsations generating a regular positive deflection wave, and IRV generating a continuous, smooth negative deflection wave (Fig. 3a). As renal venous congestion increases, venous pulsations become visible. In mildly abnormal waveforms, venous pulsations with clearly visible anterograde S and D waves are present (Fig. 3b). Severely abnormal waveforms display a retrograde S wave with only an anterograde D wave [17] (Fig. 3c).

### uJVP protocol

**Fig. 4:**
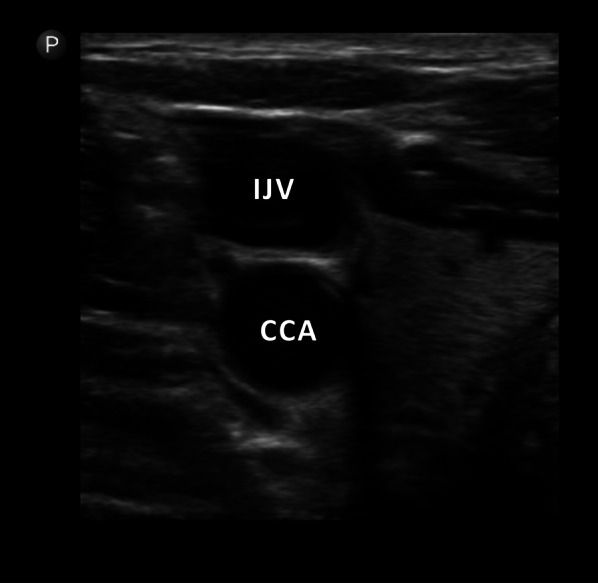
In the transverse view, the IJV can be visualized alongside the CCA; *IJV* Internal Jugular Vein. *CCA* Common Carotid Artery

**Fig. 5:**
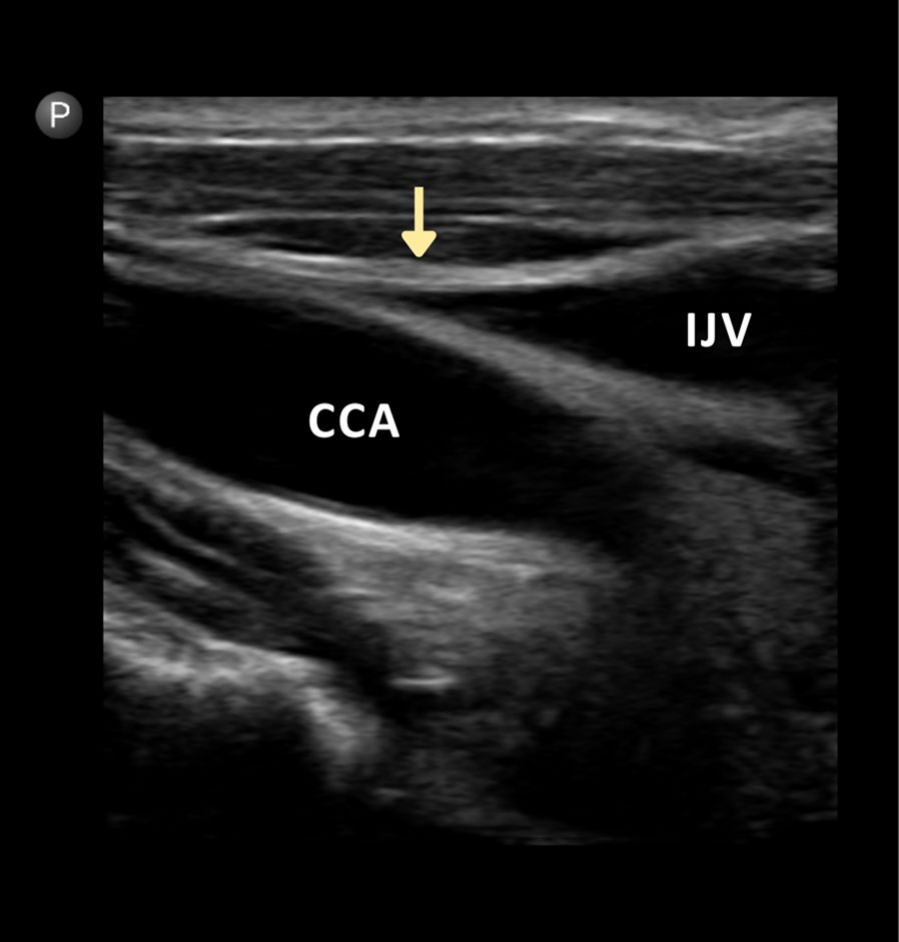
The neck bottle sign, which indicates the point of collapse in the IJV (arrow); *IJV* Internal Jugular Vein, *CCA* Common Carotid Artery

The uJVP was performed using the Philips Lumify ultrasound system with a high-frequency probe. Patients were positioned in a reclined position with the head of the bed elevated at 45 degrees. The ultrasound probe was initially placed between the two heads of the sternocleidomastoid muscle, with the probe marker pointing towards the patient's right side. In the transverse view, the internal jugular vein could be visualized alongside the common carotid artery (Fig. 4). If the internal jugular vein was not clearly visible and appeared collapsed at this position, the head of the bed was adjusted to a lower angle of around 15–30 degrees, and the visualization was rechecked. The probe was then slid cephaladly until the internal jugular vein collapsed, and the probe marker was rotated to point towards the patient's head. The goal was to locate the neck bottle sign, which indicated the point of collapse in the internal jugular vein (Fig. 5). This area was marked on the patient’s neck, and the jugular venous pressure was measured by determining the height from this point to the sternal angle in centimeters of H2O. To obtain the actual jugular venous pressure, 5 cm was added to this measured distance [14].

### Conventional VExUS grading system (VExUS)

VExUS grade 0 is assigned when the maximum IVC diameter is ≤ 2 cm. VExUS grade 1 is assigned when the maximum IVC diameter is > 2 cm and there is a combination of normal or mildly abnormal waveforms without any severely abnormal waveforms in HV, PV, and IRV. VExUS grade 2 is given when the maximum IVC diameter is > 2 cm and there is one severely abnormal waveform in either HV, PV, or IRV. VExUS grade 3 is assigned when the maximum IVC diameter is > 2 cm and there is ≥ 2 severely abnormal waveforms in HV, PV, or IRV [17].

### Newly introduced modified VExUS grading system (mVExUS)

In our newly introduced mVExUS classification system, mVExUS Grade 0 is assigned when the maximum IVC diameter is < 2 cm with CI of ≥ 50%; mVExUS Grade 1 is designated for cases where the maximum IVC diameter is < 2 cm and CI is < 50% or the maximum IVC diameter is ≥ 2 cm and CI is ≥ 50% and there is a combination of normal or mildly abnormal waveforms without any severely abnormal waveforms in HV, PV, or IRV; mVExUS Grade 2 is given when the maximum IVC diameter is < 2 cm and CI is < 50% or the maximum IVC diameter is ≥ 2 cm and CI is ≥ 50% and there is one severely abnormal waveform in either HV, PV, or IRV; and mVExUS Grade 3 is assigned when the maximum IVC diameter is < 2 cm and CI is < 50% or the maximum IVC diameter is ≥ 2 cm and CI is ≥ 50% and there are two or more severely abnormal waveforms in HV, PV, or IRV.

### IVC classification by American society of echocardiography

The American Society of Echocardiography classifies the IVC into three groups: Class 1 is designated when the maximum diameter of these IVC is ≤ 2.1 cm with CI ≥ 50%. Class 2 is assigned when the maximum diameter of the IVC is ≤ 2.1 cm with CI < 50%, or when the maximum diameter of the IVC is > 2.1 cm with CI ≥ 50%. Class 3 is allocated when the maximum diameter of the IVC is > 2.1 cm with CI < 50% [12].

### Right heart catheterization

Patients underwent RHC within 4 h of the ultrasound examination, performed by a cardiologist staff member with expertise in RHC, who subsequently recorded the RAP during the RHC procedure. We employed a 6 or 7-French balloon flotation catheter, inserted via the femoral or jugular venous approach, to measure the RAP while the patient was in the supine position. For patients in sinus rhythm, the mean of the 'a' wave during end-expiration was utilized. Conversely, the pressure at the end of the QRS complex was used to represent the RAP in other cases. Importantly, the clinician who conducted the RHC was blinded to the VExUS grading, IVC diameter, CI, and uJVP measurements.

### Statistical analysis

Continuous variables were summarized as mean with standard deviation (SD) or median with interquartile range, depending on the normality of the data, which was assessed using the Shapiro–Wilk test. Categorical variables were presented as frequency and percentage. To compare the median RAP across ASE classification or VExUS grade, Kruskal–Wallis test was performed with pairwise comparison adjusted by the Bonferroni correction for multiple tests. In assessing inter-rater variability, Cohen’s kappa statistic was employed to compute the agreement among the raters for each variable. Spearman’s correlation coefficient was calculated to assess the correlation between RAP and uJVP, and the Bland–Altman method was employed to visually evaluate their agreement. Statistical analysis was performed using Stata, version 18.0 (StataCorp).

## Results

Between September 2022 and July 2023, a total of 113 patients were initially scheduled for RHC. However, 30 patients refused to participate in the research. Out of the remaining 83 patients, 10 were excluded from the study: 9 were excluded for case cancellation and in 1 patient the hepatic vein was unable to be identified. Consequently, a total of 73 patients were included in the study. The mean age of the participants was 57 years. Female patients accounted for 41 patients (56%) in the study population. The most common reason for RHC was pulmonary hypertension (21%), followed by pre-operative evaluation (19%), and the evaluation of dyspnea etiology (16%). Among the underlying conditions, pulmonary hypertension was the most common, observed in 37 patients (51%), followed by atrial fibrillation in 25 patients (34%), and heart failure with reduced ejection fraction in 9 patients (12%). Additional baseline characteristics are shown in Table 1.The mean IVC diameter was 2.0 cm, with a mean CI of 41.9%. Of the 73 included patients, 37 (51%) were classified as VExUS grade 0, 9 (12%) as grade 1, 9 (12%) as grade 2, and 18 (25%) as grade 3. Regarding ASE classification, 16 (22%) patients were in class 1, 35 (48%) in class 2, and 22 (30%) in class 3.

**Table 1:** Cohort clinical characteristics

	N = 73^a^
Age	57 ± 17
Sex	
Male	32 (43%)
Female	41 (56%)
BMI	23 ± 4
Ejection fraction	59 ± 12
Underlying disease	
Pulmonary hypertension	37 (51%)
Atrial fibrillation	25 (34%)
Heart failure with Reduced Ejection	9 (12%)
Myocardial infarction	8 (11%)
Chronic Obstructive Pulmonary Disease	3 (4%)
Cirrhosis	2 (3%)
Tricuspid regurgitation	
Mild	27 (37%)
Moderate	13 (18%)
Severe	17 (23%)
Tricuspid stenosis	
Mild	2 (3%)
Moderate	0 (0%)
Severe	2 (3%)
Pulmonary regurgitation	
Mild	34 (47%)
Moderate	8 (11%)
Severe	1 (1%)
Pulmonary stenosis	
Mild	3 (4%)
Moderate	0 (0%)
Severe	3 (4%)
Mitral regurgitation	
Mild	30 (47%)
Moderate	3 (4%)
Severe	6 (8%)
Mitral stenosis	
Mild	1 (1%)
Moderate	2 (3%)
Severe	2 (3%)
Aortic regurgitation	
Mild	17 (23%)
Moderate	5 (7%)
Severe	0 (0%)
Aortic stenosis	
Mild	1 (1%)
Moderate	1 (1%)
Severe	0 (0%)
Congenital heart disease	23 (32%)
Reason for Right heart catheterization	
Pulmonary hypertension	15 (21%)
Pre-operative organ transplant	14 (19%)
Evaluation for dyspnea	12 (16%)
Evaluation for valvular heart disease	12 (16%)
Congenital heart disease evaluation	12 (16%)
Others	8 (12%)

Median (IQP); *n* (%)

### RAP assessment by ultrasound findings

**Fig. 6:**
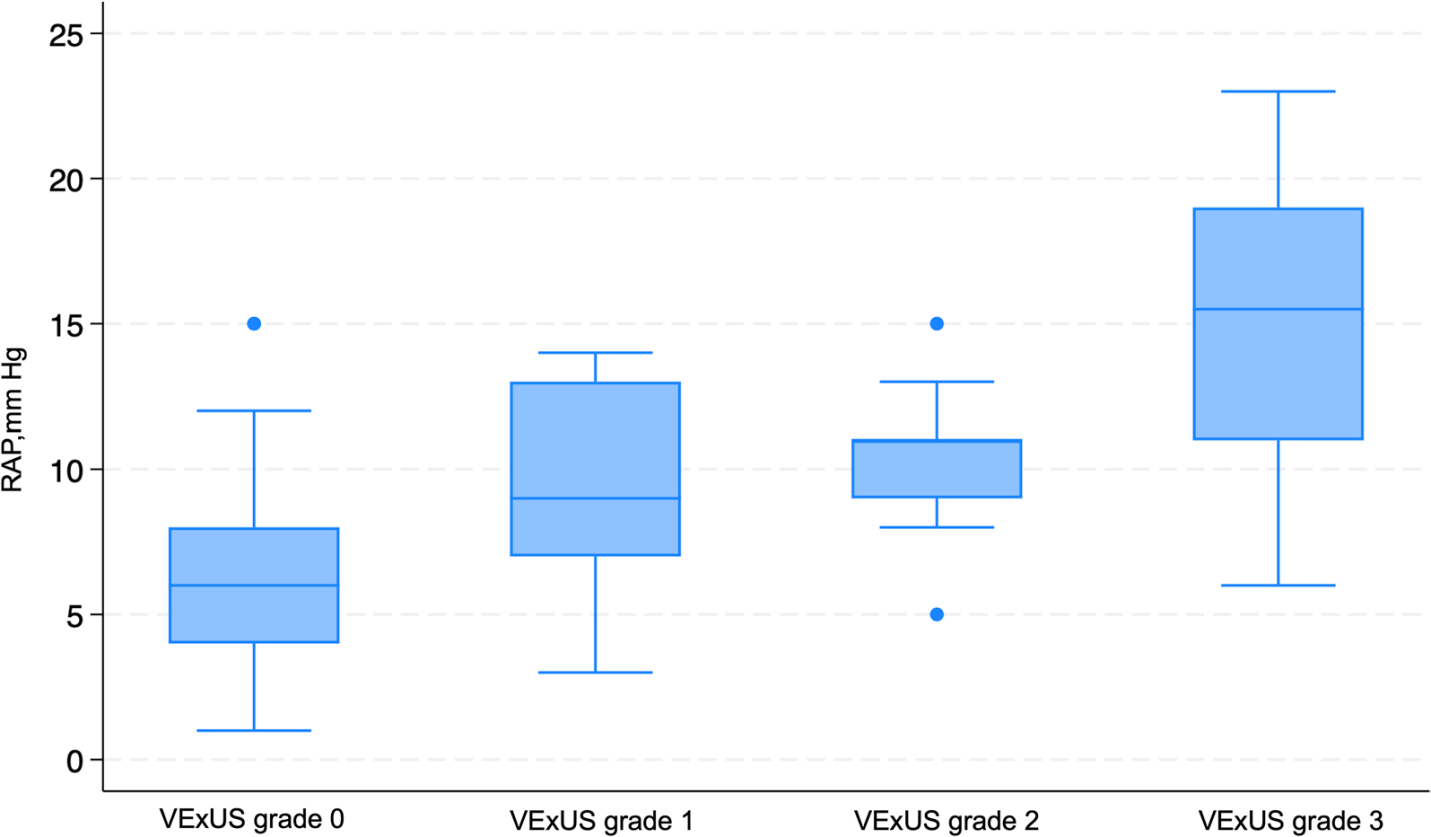
Box plot of VExUS grading and RAP measured in mmHg on RHC; *RAP* Right Atrial Pressure, *VExUS* Venous Excess Ultrasound

RAP assessment by RHC was compared across the VExUS grading. The median RAP was significantly different across VExUS grading, with the median RAP of 6 mmHg (IQR 4–8 mmHg), 9 mmHg (IQR 7–13 mmHg), 11 mmHg (IQR 9–11 mmHg), and 15.5 mmHg (IQR 11–19 mmHg) in VExUS grade 0, 1, 2, and 3, respectively (*P* < 0.001) (Fig. 6: Box plot of VExUS grading and RAP measured in mmHg on RHC). Upon conducting a post-hoc analysis, it was evident from the pairwise comparisons that there was a significant difference in RAP when comparing VExUS grade 0 to VExUS grade 3 exclusively (*P* < 0.001) (See Additional file 1).

**Fig. 7:**
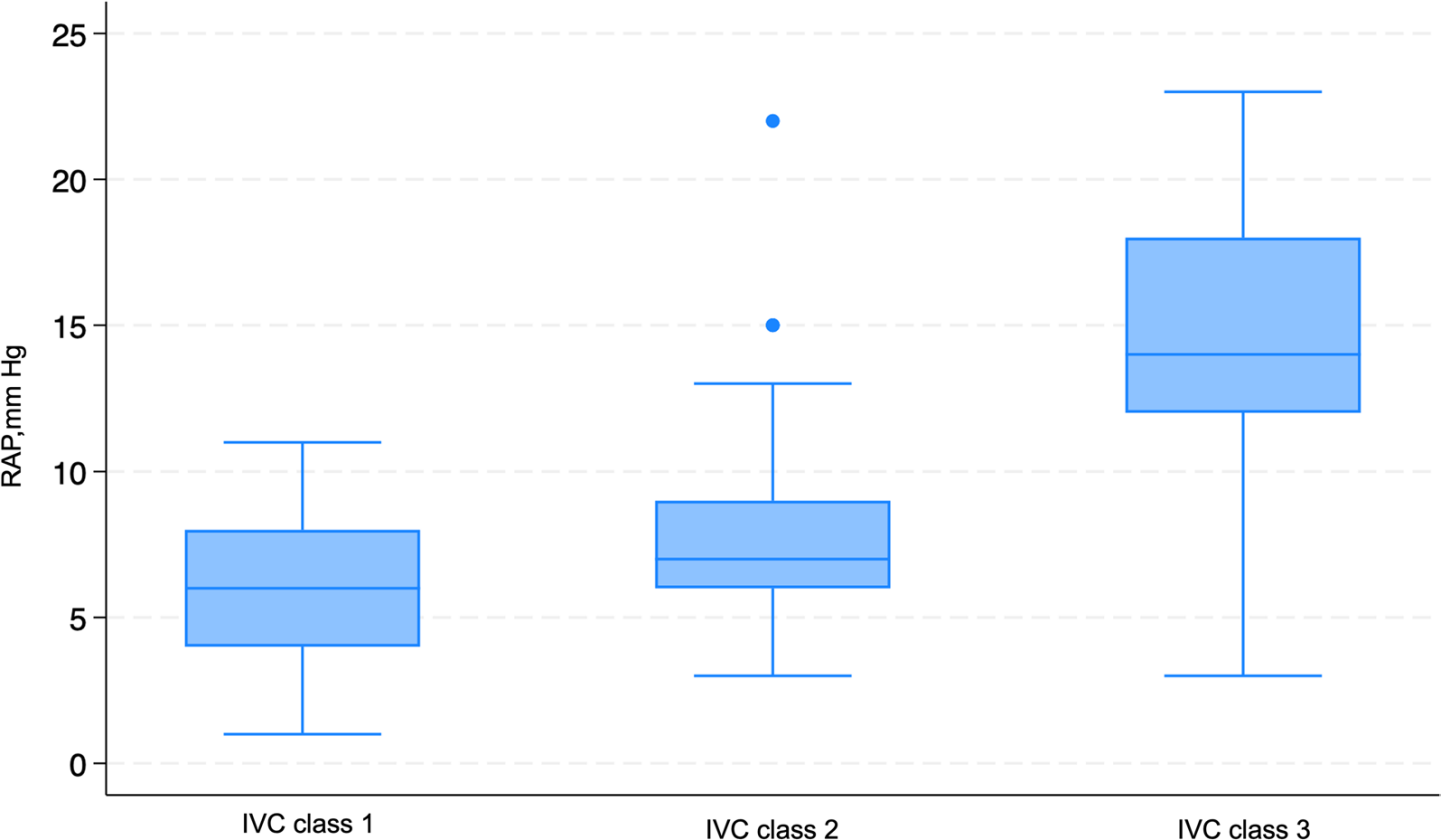
Box plot of the IVC classification by ASE with RAP measured in mmHg on RHC; *ASE* American Society of Echocardiography, *IVC* Inferior Vena Cava, *RAP* Right Atrial Pressure, *RHC* Right Heart Catheterization

**Fig. 8:**
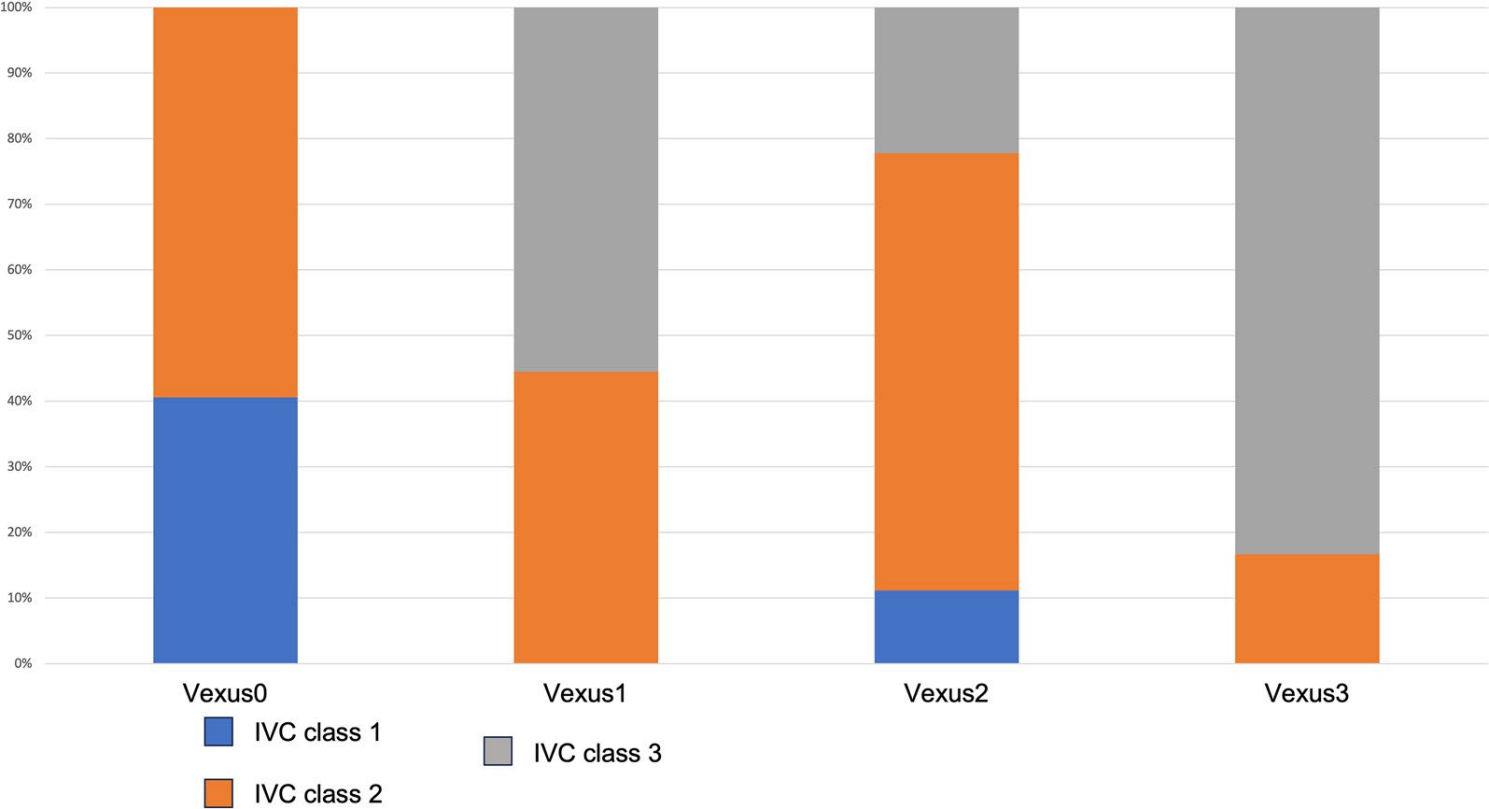
Stacked bar graph of comparison of IVC ASE classifications with VExUS grading; *ASE* American Society of Echocardiography, *IVC* Inferior Vena Cava, *VExUS* Venous Excess Ultrasound

**Fig. 9:**
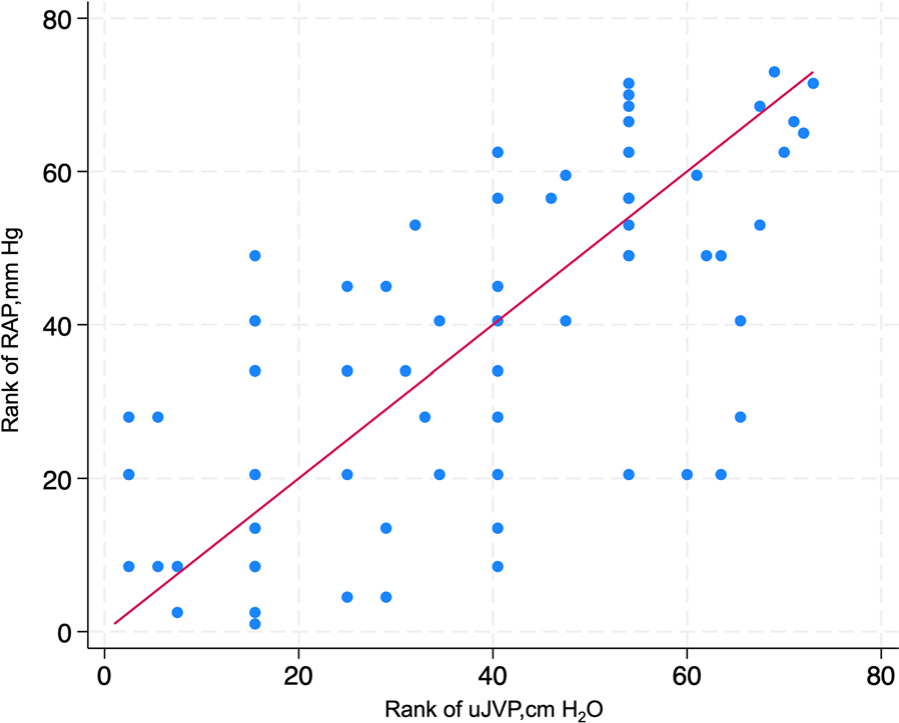
Spearman’s correlation coefficient between the rank of uJVP and the rank of RAP measured in mmHg on RHC; *RAP* Right Atrial Pressure, *RHC* Right Heart Catheterization, *uJVP* ultrasound-measured Jugular Venous Pressure

**Fig. 10:**
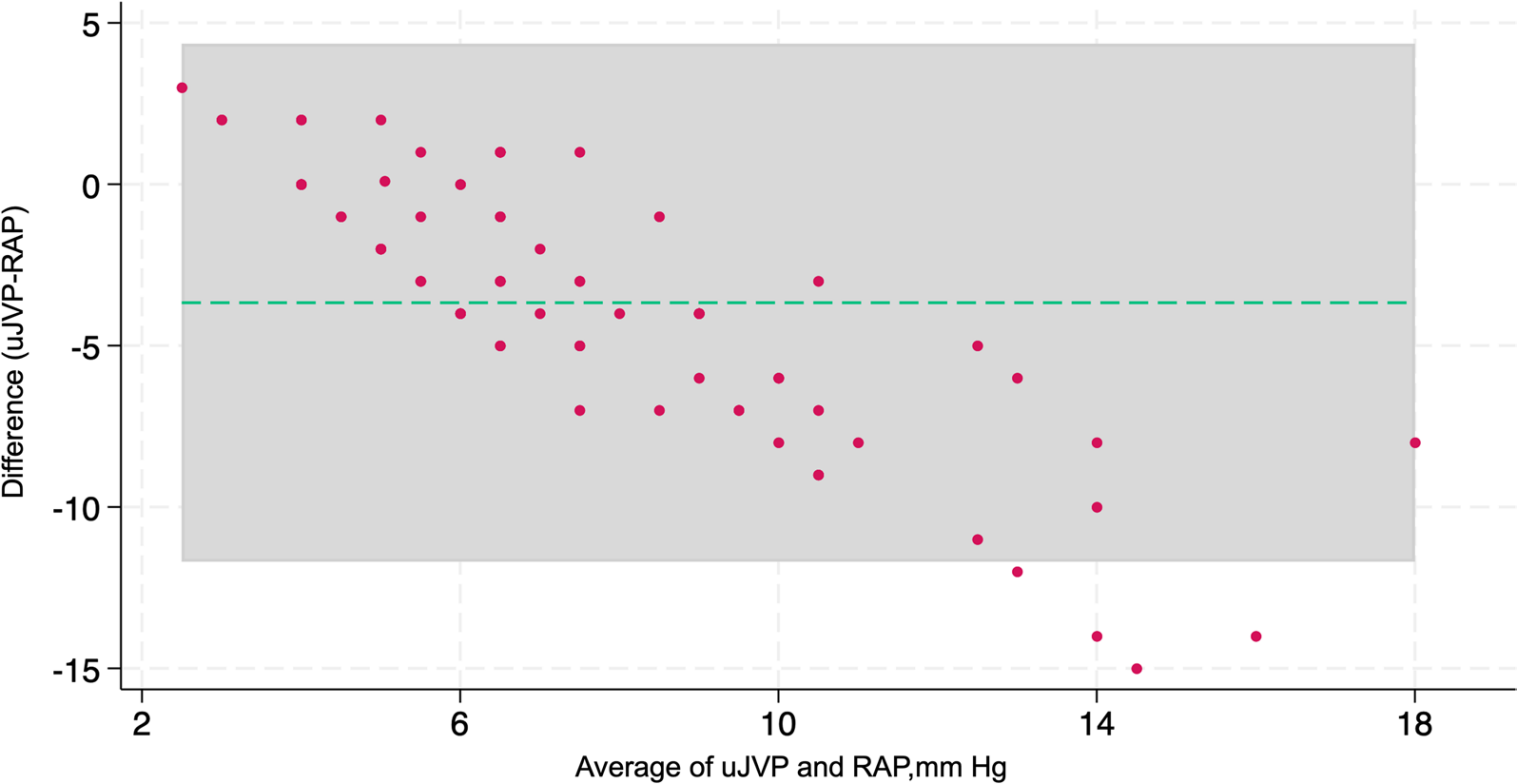
Bland–Altman Plot of uJVP and RAP; *uJVP* ultrasound-measured Jugular Venous Pressure, *RAP* Right Atrial Pressure

In the comparison RAP across IVC classification by the ASE, it was observed that the median RAP was significantly different across the IVC classification by ASE (*P* < 0.001). The median RAP was 6 mmHg among patients classified as class 1, 7 mmHg in class 2, and 14 mmHg in class 3 (Fig. 7: Box plot of the IVC classification by ASE with RAP measured in mmHg on RHC). After the primary analysis, a post-hoc examination unveiled notable disparities in RAP solely among specific classifications of IVC guided by the ASE guideline. Particularly, the RAP exhibited significant divergence in comparisons between IVC classifications denoted as per ASE guideline class 1 and 3, as well as 2 and 3 (*P* < 0.001) (See Additional file 1). In the comparison of IVC classifications with VExUS grading, the results revealed the following associations: For IVC Class 1, 15 patients (93.75%) were categorized as VExUS Grade 0, and 1 patient (6%) was categorized as VExUS Grade 2. Within IVC Class 2, the distribution comprised 22 patients (63%) in VExUS Grade 0, 4 patients (11%) in VExUS Grade 1, 5 patients (14%) in VExUS Grade 2, and 3 patients (9%) in VExUS Grade 3. Regarding IVC Class 3, the connections included 5 patients (23%) in VExUS Grade 1, 2 patients (9%) in VExUS Grade 2, and 15 patients (68%) in VExUS Grade 3 (Fig. 8: Stacked bar graph of comparison of IVC ASE classifications with VExUS grading). A comprehensive investigation into uJVP unveiled a robust and significant positive correlation with invasively measured RAP, (ρ = 0.67) and carried substantial significance (*P* < 0.001) (Fig. 9: Spearman’s correlation coefficient between the rank of uJVP and the rank of RAP measured in mmHg on RHC). Additionally, the Bland–Altman method was utilized to assess the agreement between these measures. The results of the Bland–Altman analysis are presented in Fig. 10 (Bland–Altman Plot of uJVP and RAP). The analysis showed a mean difference (bias) of −3.670 mmHg, with 95% limits of agreement ranging from −11.694 to 4.354 mmHg. This indicates that while there was a good bias, 5.48% (4 out of 73) of the measurements were outside the limits of agreement. The broad limits of agreement suggest variability between the two measurement methods, especially considering that the average values lie between 2.5 and 18 mmHg. In the newly introduced modified VExUS grading system (mVExUS) with the combination of the IVC CI, the distribution of grades from VExUS to mVExUS changed as follows: grade 0 decreased from 37 to 16 patients, grade 1 increased from 9 to 26 patients, grade 2 decreased from 9 to 13 patients, and grade 3 remained the same.

**Fig. 11:**
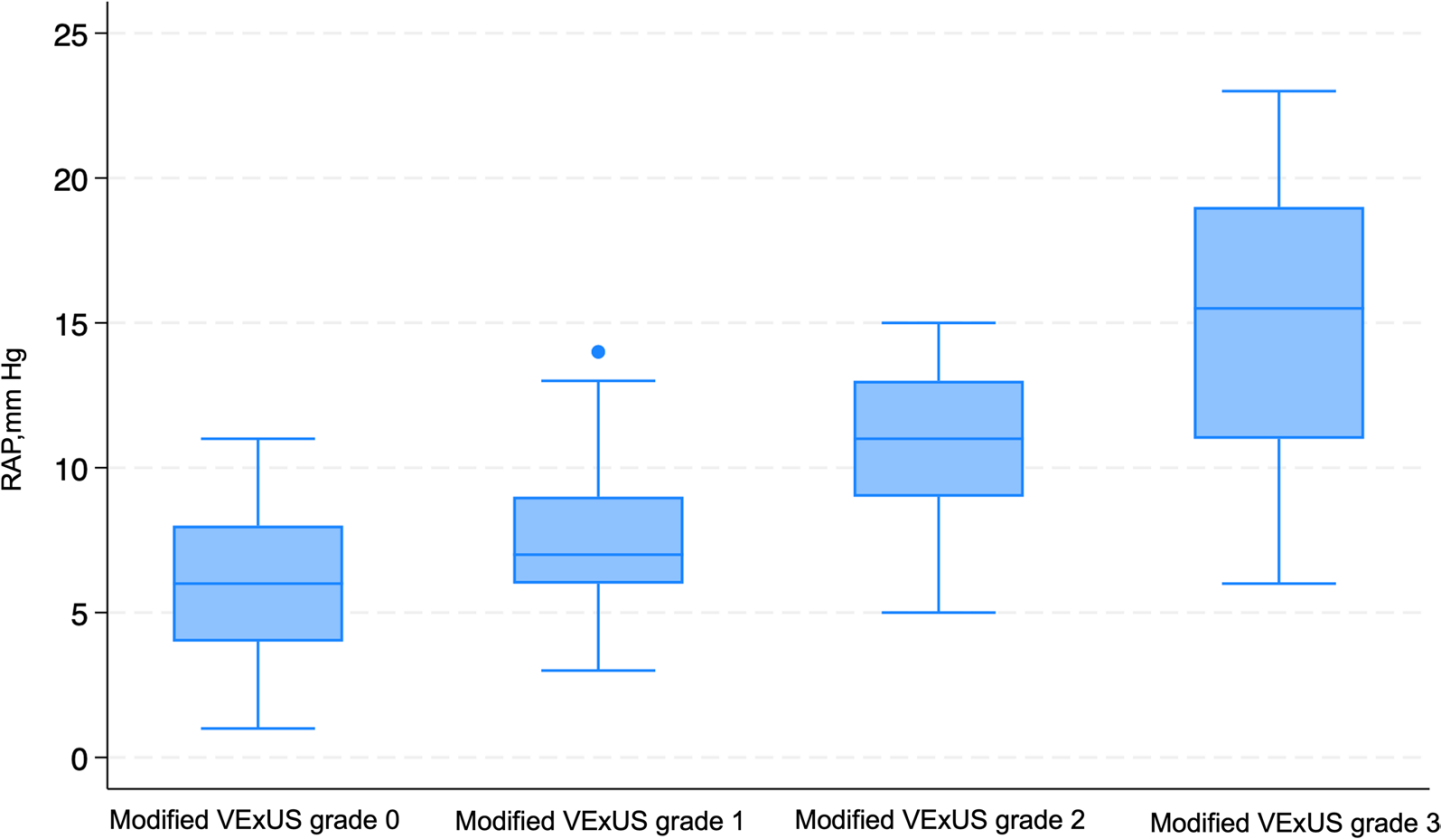
Box plot of mVExUS grading and RAP measured in mmHg on RHC; *mVExUS* Modified Venous Excess Ultrasound, *RAP* Right Atrial Pressure, *RHC* Right Heart Catheterization

In the context of the mVExUS grading system combining with the IVC CI, we observed a notable shift in the distribution of mVExUS grades correlating with RAP. Specifically, mVExUS grade 0 exhibited a median RAP of 6 mmHg, (IQR 4–8 mm Hg). For grade 1, the median RAP was 7 mmHg (IQR 6–9 mm Hg). Grade 2 displayed a median RAP of 11 mmHg (IQR 9–13 mmHg). Remarkably, mVExUS grade 3 remained unchanged from the conventional VExUS grading system with median RAP of 15.5 mmHg (Fig. 11: Box plot of mVExUS grading and RAP measured in mmHg on RHC). Intriguingly, when we conducted a comparative analysis of RAP among these groups, we uncovered a significant difference (*P* < 0.001). Delving deeper into post-hoc analysis, we identified statistically significant differences between group 0 and group 3 (*P* < 0.001), as well as between group 1 and group 3 (*P* < 0.001), and between group 0 and group 2 (*P* = 0.019) (see Additional file 1).

## Discussion

### Correlation with RAP

Our study confirms a significant correlation between venous ultrasound measurements (VExUS, IVC, and uJVP) and RAP, as determined by RHC. Notably, this correlation is particularly strong in patients with severe venous congestion (VExUS grade 3). RAP showed significant differences among the VExUS grades; however, post-hoc analysis revealed that the RAP was significantly different only between VExUS grade 0 and 3. The analysis revealed a significant difference in median RAP across IVC classifications by the ASE, particularly between class 1 and 3, as well as class 2 and 3. In the newly introduced modified VExUS grading system (mVExUS), which incorporates the IVC CI, the distribution of VExUS grades changed as follows: grade 0 and 2 decreased, grade 1 increased, while grade 3 remained unchanged. In the context of mVExUS, incorporating the IVC CI, we observed distinct shifts in mVExUS grade distribution correlated with RAP. Elevated mVExUS grade appears to be associated with greater RAP. A comparative analysis of RAP among these groups unveiled significant differences. Specifically, significant differences were found between group 0 and group 3, group 1 and group 3, as well as between group 0 and group 2.

### Comparison with previous studies

Our findings align with Longino et al.'s pilot study, emphasizing the positive correlation between RAP and VExUS grading [18]. In contrast to the pilot study, our larger sample size included 18 patients with VExUS grade 3, offering valuable insights into this less common grade. Additionally, we extended our analysis to compare the VExUS grading system with IVC diameter, including its CI, and uJVP in relation to RAP.

### VExUS grade discrepancy

In our study, the conventional VExUS grades 0, 1, and 2 did not significantly predict RAP. This divergence can be attributed to the original VExUS criteria, which solely considered an IVC measurement of > 2.0 cm and did not incorporate the CI for classification. Conversely, the ASE's IVC classification leverages the CI for patient stratification. This discrepancy clarifies why grades 0, 1, and 2 could not effectively differentiate patients in the original VExUS grading system. However, our mVExUS grading system improved discrimination between grades 0 and 2, as well as grades 1 and 3. We introduced the CI due to potential challenges related to IVC size accuracy in Asian populations, where a consistent IVC size of 2 cm may not apply universally. Additionally,, adhering to the ASE guideline, we observed that a CI of less than 50% correlated with increased RAP. Importantly, these results cannot be applied to patients on mechanical ventilation (invasive or non-invasive with positive end-expiratory pressure) and in patients with respiratory distress, where the CI could be affected by increased intra-thoracic pressure shifts [20].

### IVC equivalence

Our comparisons between IVC and the conventional VExUS grading system indicated that VExUS grade 0 may be analogous to IVC class 1, supported by the similar median values of RAP in these categories. Likewise, there were similarities between VExUS grade 3 and IVC class 3, mirroring the correlation found between VExUS grade 0 and IVC class 1. However, medians for VExUS grade 1 and 2 did not align with IVC class 2. Further research is needed to investigate the relationships between VExUS grade 1, 2, and IVC class 2 for improved RAP prediction in the future.

### Ultrasound JVP implications

Extensive research on ultrasound-measured uJVP has unveiled a robust and highly significant positive correlation with invasively measured RAP, highlighting the reliability and clinical relevance of uJVP as a valuable non-invasive tool for assessing right atrial pressure in various medical contexts. When aligned with the research conducted by Libo Wang et al., our findings regarding the correlation of uJVP with RAP were consistent, further reinforcing the robustness of this association [12]. Although the Bland–Altman analysis shows a good bias, the broad limits of agreement suggest that the measurements from the two methods are not consistently reliable. This inconsistency may affect the practical application of these methods. Specifically, while there is overall good agreement between uJVP and RAP, the limits of agreement widen at higher RAP values, indicating potentially less consistent agreement in patients with very high RAP. This widening of the limits of agreement may suggest an underestimation of RAP by uJVP at elevated pressures.

### Clinical implications, limitations, and future research

Our findings have several important clinical implications. First, the correlation between non-invasive ultrasound measurements and RAP suggests that these techniques can be valuable tools for risk assessment and management of patients at risk of venous congestion. Secondly, the modified VExUS grading system, which incorporates IVC CI, offers a promising approach to better stratify patients based on their risk of elevated RAP.

It is essential to acknowledge the limitations of our study. First, we excluded mechanically ventilated and critically ill patients, which may introduce selection bias and limit the generalizability of our findings. Additionally, our study was conducted in an outpatient setting, and the sample size for certain VExUS grades (1 and 2) was limited. Future research should aim to address these limitations and explore the applicability of these techniques in broader clinical contexts. These findings should not be extended to patients receiving mechanical ventilation (both invasive and non-invasive with positive end-expiratory pressure) or those experiencing respiratory distress, as intra-thoracic pressure fluctuations may impact the CI [20]. In assessing the uJVP, adjustments to the headboard angle between 15° and 30° were made for reassessment if the vein appeared collapsed at 45°. The variability in headboard angulation among patients raises a question regarding its potential influence on the obtained values, which could impact the correlation results with RAP. Therefore, it may be beneficial to acknowledge this variability and its potential impact in the study limitations. We acknowledge the limitation of not having conducted a formal sample size calculation for this study. The absence of a specific reference paper directly applicable to our research context poses a potential limitation to our study design. To address this in future studies, we will ensure that sample size calculations are based on relevant research findings.

## Conclusion

This study demonstrates a strong correlation between non-invasive ultrasound measurements, including VExUS, IVC diameter and CI, and uJVP with invasively measured RAP in an Asian population. These findings highlight the potential clinical utility of these non-invasive techniques in assessing venous congestion and risk-stratifying patients. Further research is needed to refine and validate these approaches in larger and more diverse patient cohorts, ultimately enhancing their role in cardiovascular assessment and patient management.

## Supplementary Information

Supplementary material 1. Appendix of additional information including, validation of ultrasound, protocol flow chart and table of pairwise comparison.

Supplementary material 2. Glossary of definitions for data extracted from electronic medical record.

## Data Availability

The data used and/or analyzed during the current study are available from the corresponding author on reasonable request.

## Abbreviations

RHC: Right heart catheterization
VExUS: Venous excess ultrasound
IVC: Inferior vena cava
CI: Collapsibility index
uJVP: Ultrasound-measured jugular venous pressure
RAP: Right atrial pressure
ASE: American Society of Echocardiography
HV: Hepatic vein
PV: Portal vein
IRV: Interlobar renal vein
IJV: Internal jugular vein
CCA: Common carotid artery
