# Supplementary material for: Correlation between right atrial pressure measured via right heart catheterization and venous excess ultrasound, inferior vena cava diameter, and ultrasound-measured jugular venous pressure: a prospective observational study
Source: Ultrasound J. 2024 Nov 29;16:50. doi: 10.1186/s13089-024-00397-y (PMC11607288; doi:10.1186/s13089-024-00397-y)
Supplement: Supplementary file 1 — Supplementary material 1. Appendix of additional information including, validation of ultrasound, protocol flow chart and table of pairwise comparison. [file 13089_2024_397_MOESM1_ESM.docx]

**Supplement 1**

Inter-rater reliability for ultrasound

To assess inter-rater reliability, a subset of 20 variables from 20 randomly selected patients was assessed by two members of the research team. Cohen's kappa statistic was calculated for each variable to evaluate agreement between the raters.

| **Variable** | **Kappa Statistic** | **P-value** |
| --- | --- | --- |
| Ultrasound IVC | 0.7467 | 0.0000 |
| VExUS | 1.0000 | 0.0000 |
| uJVP | 0.9381 | 0.0000 |

Table 1: Kappa statistics used in the inter-rater reliability assessment of Ultrasound between examiners, the Kappa result can be interpreted as follows: values ≤ 0 indicate no agreement, 0.01–0.20 suggest none to slight agreement, 0.21–0.40 indicate fair agreement, 0.41–0.60 imply moderate agreement, 0.61–0.80 represent substantial agreement, and 0.81–1.00 denote almost perfect agreement (1).

Patient Flow Diagram

**113 patients screened for enrollment**

**30 patients did not meet inclusion criteria**

**83 patients underwent VExUS examination**

**73 patients were included**

**1 patient was excluded due to poor image quality**

**9 patients were excluded due to case cancellations**

Pairwise comparisons

| Sample1-Sample2 | Test statistic | Std. Error | Std. Test Statistic | Sig. | Adj. Sig.^a^ |
| --- | --- | --- | --- | --- | --- |
| 0-1 | -14.087 | 7.836 | -1.792 | 0.073 | 0.439 |
| 0-2 | -18.698 | 7.863 | -2.378 | 0.017 | 0.104 |
| 0-3 | -31.726 | 6.080 | -5.218 | 0.000 | 0.000 |
| 1-2 | -4.611 | 9.973 | -0.462 | 0.644 | 1.000 |
| 1-3 | -17.639 | 8.637 | -2.042 | 0.041 | 0.247 |
| 2-3 | -13.028 | 8.837 | -1.508 | 0.131 | 0.789 |

Table 1: Pairwise comparisons of VExUS

| Sample1-Sample2 | Test statistic | Std. Error | Std. Test Statistic | Sig. | Adj. Sig.^a^ |
| --- | --- | --- | --- | --- | --- |
| 1-2 | -9.627 | 6.384 | -1.508 | 0.395 | 0.439 |
| 1-3 | -33.835 | 6.951 | -4.868 | 0.000 | 0.104 |
| 2-3 | -24.208 | 5.756 | -4.206 | 0.000 | 0.000 |

Table 2: Pairwise comparisons of IVC

| Sample1-Sample2 | Test statistic | Std. Error | Std. Test Statistic | Sig. | Adj. Sig.^a^ |
| --- | --- | --- | --- | --- | --- |
| 0-1 | -6.745 | 6.722 | -1.003 | 0.316 | 1.000 |
| 0-2 | -23.264 | 7.899 | -2.945 | 0.003 | 0.019 |
| 0-3 | -35.049 | 7.269 | -4.822 | 0.000 | 0.000 |
| 1-2 | -16.519 | 7.186 | -2.299 | 0.022 | 0.129 |
| 1-3 | -28.303 | 6.487 | -4.363 | 0.000 | 0.000 |
| 2-3 | -11.784 | 7.700 | -1.530 | 0.126 | 0.756 |

Table 3: Pairwise comparisons of modified VExUS

Each row tests the null hypothesis that Sample 1, and Sample 2 distributions are the same.

Asymptomatic significances (2-sided tests) are displayed. The significance level is 0.05.

1. Significance values have been adjusted by the Bonferroni correction.

Reference:

1. McHugh M. Interrater reliability: The kappa statistic. Biochemia medica : časopis Hrvatskoga društva medicinskih biokemičara / HDMB. 2012;22:276-82.<https://doi.org/10.11613/BM.2012.031>.
